# TSLP regulates mitochondrial ROS-induced mitophagy via histone modification in human monocytes

**DOI:** 10.1186/s13578-022-00767-w

**Published:** 2022-03-15

**Authors:** Yi-Ching Lin, Yu-Chih Lin, Mei-Lan Tsai, Wei-Ting Liao, Chih-Hsing Hung

**Affiliations:** 1 Department of Laboratory Medicine, Kaohsiung Medical University Hospital, Kaohsiung Medical University, Kaohsiung, Taiwan; 2 Doctoral Degree Program in Toxicology, College of Pharmacy, Kaohsiung Medical University, Kaohsiung, Taiwan; 3 Department of Laboratory Medicine, School of Medicine, College of Medicine, Kaohsiung Medical University, Kaohsiung, Taiwan; 4 Department of Medical Research, Kaohsiung Medical University Hospital, Kaohsiung Medical University, Kaohsiung, Taiwan; 5 Division of General Internal Medicine, Department of Internal Medicine, Kaohsiung Medical University Hospital, Kaohsiung Medical University, Kaohsiung, Taiwan; 6 Department of Medical Humanities and Education, School of Medicine, College of Medicine, Kaohsiung Medical University, Kaohsiung, Taiwan; 7 Graduate Institute of Medicine, College of Medicine, Kaohsiung Medical University, Kaohsiung, Taiwan; 8 Department of Pediatrics, School of Medicine, College of Medicine, Kaohsiung Medical University, Kaohsiung, Taiwan; 9 Department of Biotechnology, College of Life Science, Kaohsiung Medical University, No. 100 Shih-Chuan 1st Road, Kaohsiung, 807 Taiwan; 10 Research Center for Environmental Medicine, Kaohsiung Medical University, Kaohsiung, Taiwan; 11 Department of Pediatrics, Kaohsiung Municipal Siaogang Hospital, Kaohsiung, Taiwan; 12 Department of Pediatrics, Kaohsiung Medical University Hospital, Kaohsiung Medical University, #100, Tz-You 1st Road, Kaohsiung, 807 Taiwan, ROC

**Keywords:** TSLP, Reactive oxygen species (ROS), Mitophagy, Histone modification, Chemokine, Monocytes

## Abstract

**Background:**

Thymic stromal lymphopoietin (TSLP) is a Th2-like cytokine involved in asthma pathogenesis. Excessive reactive oxygen species (ROS) production can lead to airway inflammation, hyperresponsiveness and remodeling. Mitophagy, followed by ROS production, is the selective degradation of mitochondria by autophagy and often occurs in defective mitochondria. In the present study, we aimed to examine the effects of TSLP on ROS production and mitophagy in human monocytes and to investigate the underlying mechanisms, including epigenetic regulation.

**Results:**

TSLP induced ROS generation, and the effects were reversed by the antioxidant N-acetylcysteine (NAC) in THP-1 cells. Transmission electron microscopy images showed donut-shaped mitochondria that lost the cristae ultrastructure after TSLP stimulation. A decrease in mitochondrial membrane potential, decreased MTCO2 expression, and increased mitochondrial DNA release after TSLP stimulation were found. TSLP enhanced mitochondrial complex I and complex II/III activity and increased mitochondrial copy numbers and the expression of the complex II *SHDA* gene. TSLP-induced *SHDA* expression was inhibited by the histone acetyltransferase inhibitor anacardic acid (AA) and the histone methyltransferase inhibitor methylthioadenosine (MTA), and chromatin immunoprecipitation assays revealed that TSLP enhanced H3 acetylation, H4 acetylation, and H3K4 and H3K36 trimethylation in the *SHDA* promoter. Confocal laser microscopy showed that TSLP treatment increased the signals of the mitophagy-related proteins PINK1, LC3, phospho-parkin and phospho-ubiquitin, and pretreatment with AA and MTA reduced TSLP-induced PINK1 and LC3 accumulation in mitochondria. Western blot analysis showed that TSLP significantly increased phosphor-AMPK signal intensity, and the effects were inhibited by the antioxidant NAC. The increased signal intensities of the mitophagy-related proteins PINK1, Parkin and LC3 I/II were decreased by dorsomorphin, an AMPK inhibitor. TSLP decreased M1-related cytokine CXCL-10 production and increased M2-related cytokine CCL-1 and CCL-22 production, which was suppressed by the mitophagy inhibitor Mdivi-1 and PINK1 gene knockdown.

**Conclusions:**

Epithelial-derived TSLP regulates ROS production and mitophagy through AMPK activation and histone modification and alters M1/M2 chemokine expression in human monocytes.

**Graphical Abstract:**

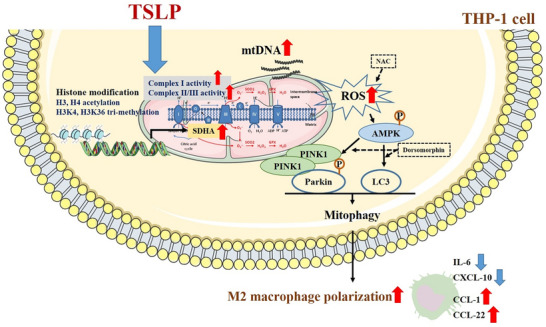

**Supplementary Information:**

The online version contains supplementary material available at 10.1186/s13578-022-00767-w.

## Background

Asthma is one of the most common chronic diseases in childhood, and its prevalence has been increasing in industrializing countries. Thymic stromal lymphopoietin (TSLP), interleukin (IL)-25 and IL-33 are upstream epithelial cytokines and play important roles in allergic diseases [1, 2]. TSLP can be produced by epithelial cells, smooth muscle cells and mast cells in allergic diseases [1]. Furthermore, TSLP drives the development of strong allergic Th2 responses and IL-4, IL-5 and IL-13 release through the upregulation of OX40 ligand expression in dendritic cells [3]. The expression of TSLP and TSLP receptors is significantly higher in asthmatic children than in nonasthmatic children [4]. The exhaled breath condensate levels of TSLP are significantly higher in patients with asthma than in controls [5]. TSLP overexpression in the airways of asthmatic patients has been reported, and TSLP polymorphisms are linked to asthma susceptibility [6, 7].

Oxidative stress, which is physiological damage due to reactive oxygen species (ROS), has been demonstrated to induce smooth muscle contraction, airway hyperresponsiveness, mucus secretion, and epithelial shedding [8, 9]. Excessive ROS production induced by TGF-β may lead to the development and persistence of pulmonary fibrosis [10]. ROS are also considered vital mediators of pulmonary vascular cell proliferation by enhancing vascular endothelial growth factor expression, leading to increased pulmonary arterial wall thickness and promoting vascular remodeling in airway and pulmonary diseases [11].

Mitophagy, which is mitochondrial autophagy, is important for mitochondrial quality control and essential for providing cellular energy, calcium homeostasis, redox signaling and apoptotic signaling [12, 13]. Mitophagy often occurs in defective mitochondria following damage or stress. Exposure to high levels of ROS causes mitochondrial damage [13]. The signaling pathway mediated by PTEN-induced putative kinase 1 (PINK1) and the E3 ubiquitin ligase parkin that induces mitophagy in mammalian cells has been reported [12]. PINK1 accumulates on the outer membrane of damaged mitochondria, activates parkin activity, and recruits parkin to damaged mitochondria; then, parkin ubiquitinates outer mitochondrial membrane proteins to induce mitophagy [14]. Microtubule-associated protein 1 light-chain 3 (LC3) is one of the components in the autophagy pathway [15]. ROS-induced mitophagy in alveolar macrophages contributes to pulmonary fibrosis [16].

Histone acetylation and methylation controlled by histone acetyltransferase (HAT), histone deacetylase (HDAC), and histone methyltransferase epigenetically regulate gene expression. Acetylation of histone 3 (H3) and H4 and trimethylation of H3K4 and H3K36 are associated with gene activation [17]. Asthma is considered to be the result of complex interactions between genetic predispositions and environmental influences via epigenetic regulation during intrauterine life and infancy [18, 19].

Both TSLP and ROS production are important for the pathogenesis of asthma and other allergic diseases. However, the effects of TSLP on ROS production remain unclear. In the present study, we investigated the effects of TSLP on ROS production and mitophagy and the detailed mechanisms, including epigenetic regulation.

## Results

### TSLP induced ROS production and promoted mitochondrial complex activity in THP-1 cells

**Fig. 1:**
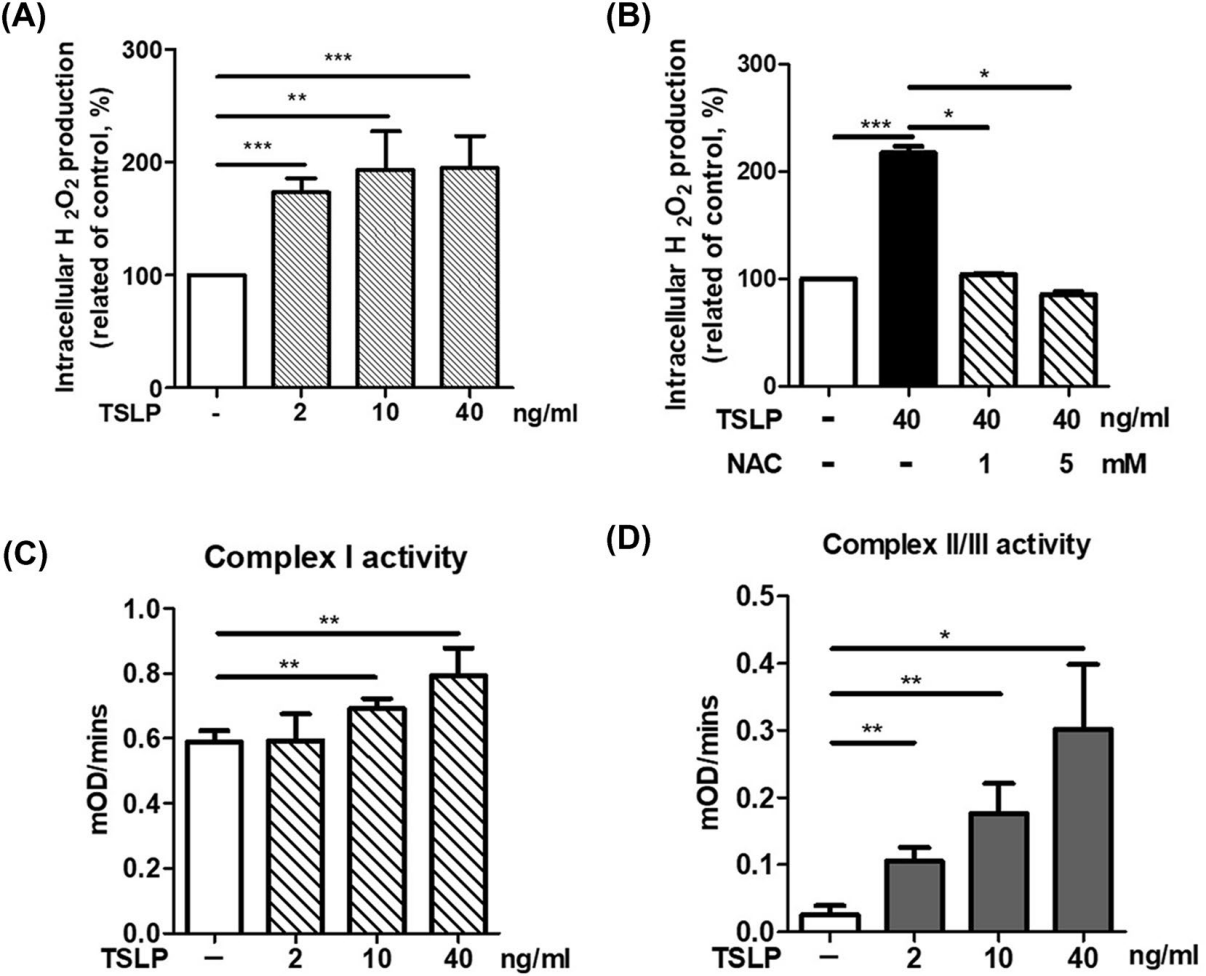
TSLP increased ROS production and mitochondrial complex activity in THP-1 cells. **A** TSLP treatment for 2 h induced ROS production in THP-1 cells. **B** Pretreatment with N-acetylcysteine (NAC), a well-established antioxidant, significantly suppressed TSLP-induced (40 ng/mL) ROS expression in THP-1 cells. **C** TSLP increased mitochondrial complex I activity in THP-1 cells. **D** TSLP increased mitochondrial complex II/III activity in THP-1 cells. Each bar represents the mean ± standard deviation of three independent experiments. *p < 0.05, **p < 0.01 and ***p < 0.001

We first investigated whether TSLP could induce ROS production in monocytes. THP-1 cells were pretreated with different concentrations of TSLP. Pretreatment with TSLP (2, 10 and 40 ng/mL) significantly increased ROS production in THP-1 cells at the 2-h time point (Fig. 1A), and the effects were suppressed by the antioxidant N-acetylcysteine (NAC) (Fig. 1B). Then, we examined the effects of TSLP on mitochondrial complex activity to investigate whether mitochondria were involved in TSLP-induced ROS production. The activities of mitochondrial complex I and complex II/III were significantly increased after 2 h of TSLP stimulation in a dose-dependent manner (Fig. 1C, D). These data indicated that mitochondria were involved in TSLP-induced ROS production.

### TSLP altered mitochondrial integrity and induced mitophagy in THP-1 cells

**Fig. 2:**
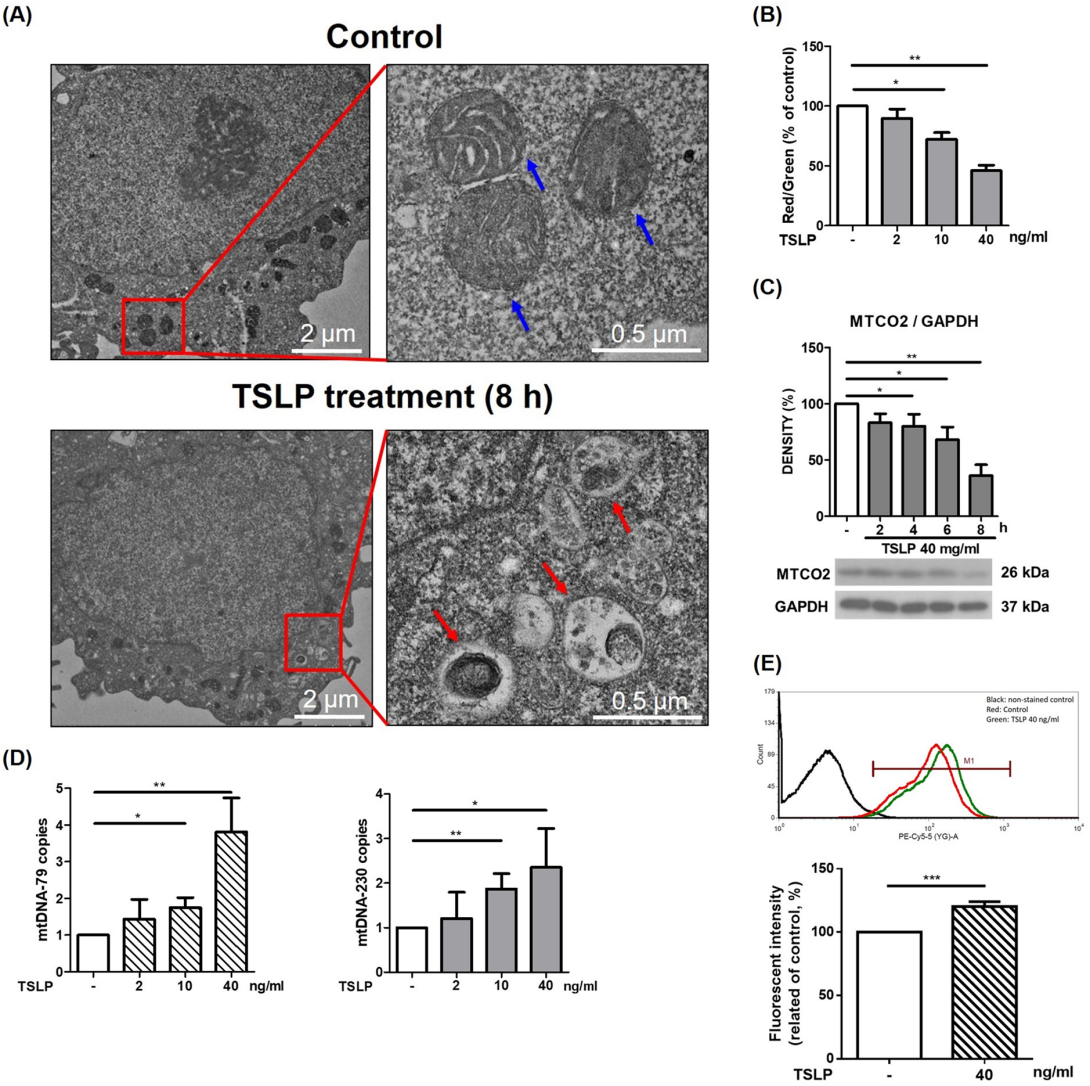
TSLP altered mitochondrial integrity and induced mitophagy in THP-1 cells. **A** Transmission electron microscopy (TEM) image of control (upper panels) and TSLP-treated THP-1 cells (lower panels). The blue arrows indicates the cristae structure of healthy mitochondria (upper right panel). The autophagic vacuoles contained damaged mitochondria-like structures in TSLP-treated cells (lower right panel, red arrows). Compared with the control group, TSLP treatment induced autophagic vacuoles in THP-1 cells. **B** The change in mitochondrial membrane potential upon TSLP treatment was measured by flow cytometry with JC-1 dye. A decrease in the red/green fluorescence intensity ratio indicated that TSLP promoted mitochondrial depolarization. **C** western blotting showed significantly decreased MTCO2 (mitochondrial inner membrane protein) expression upon TSLP treatment for 4, 6, and 8 h. **D** RT-qPCR was simultaneously performed to assess the cell-free mtDNA-79 and mtDNA-230 copies. **E** Mitophagy detection dye to quantify THP-1 cell mitophagy showed significantly increased fluorescence upon treatment with 40 ng/mL TSLP. *p < 0.05, **p < 0.01 and ***p < 0.001 between THP-1 cells with and without TSLP treatment

We performed transmission electron microscopy (TEM) analysis to observe whether TSLP alters mitochondrial integrity and induces mitophagy. We found that mitochondria decreased in size, cristae were lost, and autophagic vacuoles contained damaged mitochondria after TSLP treatment (Fig. 2A). We also investigated the change in mitochondrial membrane potential, MTCO2 (mitochondrial inner membrane protein) expression, and mitochondrial DNA (mtDNA) release of mitochondria upon TSLP treatment. A decrease in the red/green fluorescence intensity ratio indicated that TSLP promoted mitochondrial depolarization in a dose-dependent manner (Fig. 2B). Western blotting showed significantly decreased MTCO2 protein expression upon TSLP treatment for 4–8 h (Fig. 2C). Quantitative real-time PCR showed that TSLP (40 ng/mL) stimulation up-regulated mtDNA-79 (3.81 ± 0.93 folds) and mtDNA-230 (2.35 ± 0.87 folds) in THP-1 cells. TSLP increased the fraction of mitophagy-induced release of mtDNA (Fig. 2D). Furthermore, we used mitophagy detection dye to quantify THP-1 cell mitophagy by TSLP treatment. It showed significantly increased fluorescence with TSLP 40 ng/mL (Fig. 2E). All of these results indicated that TSLP altered mitochondrial integrity and promoted mitophagy in THP-1 cells.

### The effects of TSLP on the gene expression of the mitochondrial respiratory chain complex in THP-1 cells

**Fig. 3:**
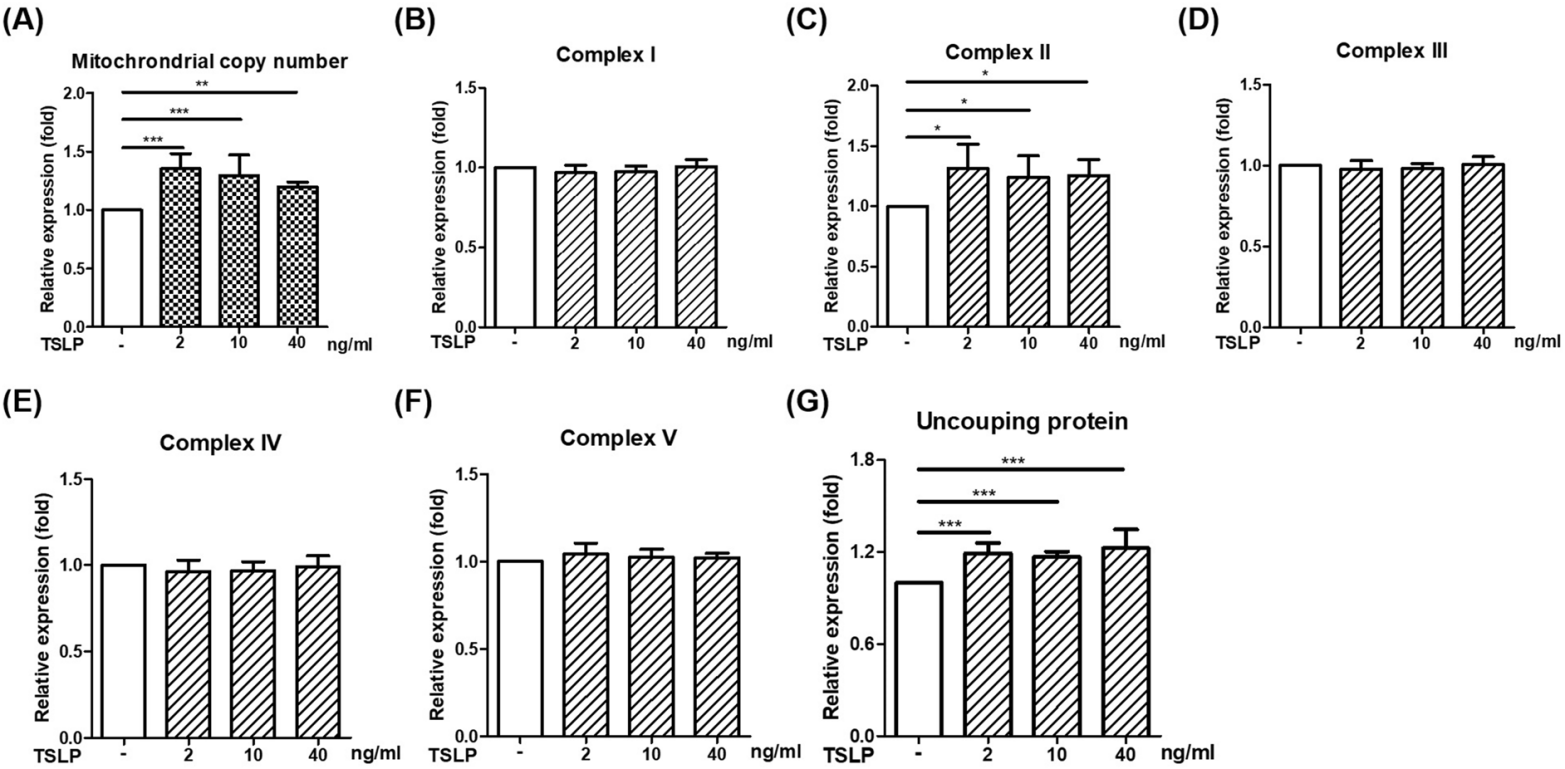
The effects of TSLP on the gene expression of the mitochondrial respiratory chain complex in THP-1 cells. TSLP significantly increased mitochondrial copy numbers, mitochondrial respiratory chain complex II *SDHA* mRNA, and uncoupling protein expression (**A**, **C**, and **G**). TSLP stimulation for 2 h did not increase the RNA expression of **B** complex I gene *ND1*, **D** complex III *CYTB*, **E** complex IV *COX1* or **F** complex V *ATP6*. The results represent the means ± standard deviation. *p < 0.05, **p < 0.01 and ***p < 0.001 between THP-1 cells with and without TSLP treatment

To investigate TSLP-induced changes in mitochondria, we performed a mitochondrial biogenesis experiment by determining mitochondrial copy number and respiratory chain complex I-V gene expression. As shown in Fig. 3, TSLP stimulation for 2 h significantly increased the mitochondrial copy numbers and uncoupling proteins (UCPs). The gene expression of *ND1* (respiratory chain complex I), *SDHA* (respiratory chain complex II), *CYTB* (respiratory chain complex III), *COX1* (respiratory chain complex IV) and *ATP6* (respiratory chain complex V) was screened using qRT–PCR, and we found that TSLP significantly increased the expression of the complex II gene *SDHA* but not the others (Fig. 3).

### TSLP promoted the expression of respiratory chain complex II *SDHA* via epigenetic regulation

**Fig. 4:**
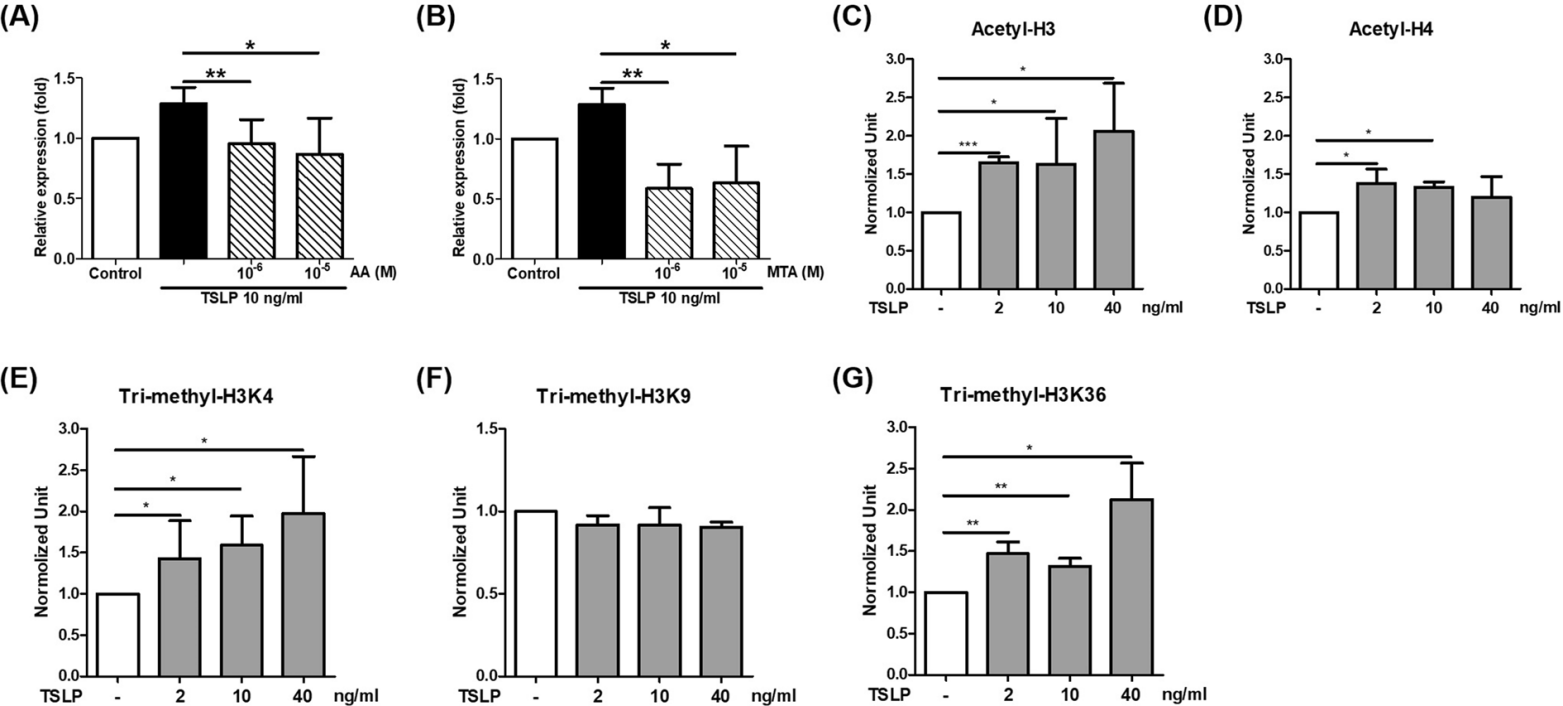
TSLP promoted respiratory chain complex II SDHA expression via epigenetic regulation. TSLP-induced *SHDA* expression was inhibited by **A** the histone acetyltransferase inhibitor anacardic acid (AA) and **B** the histone methyltransferase inhibitor methylthioadenosine (MTA). Cells were pretreated with AA or MTA for 1 h prior and then stimulated with TSLP for 2 h. ChIP analysis revealed that TSLP enhanced **C** H3 acetylation and **D** H4 acetylation in the *SHDA* promoter. TSLP also significantly enhanced H3K4 and H3K36 trimethylation (**E** and **G**) but did not enhance H3K9 trimethylation (**F**) in the *SHDA* promoter. *p < 0.05, **p < 0.01 and ***p < 0.001 between THP-1 cells with and without TSLP treatment

We investigated whether *SHDA* is involved in TSLP-induced mitophagy. The fluorescence intensity of mitophagy was significantly increased upon TSLP treatment in the nontargeting control (Additional file 1: Fig. S1). When the *SHDA* gene was knocked down, the increased fluorescence intensity of TSLP-induced mitophagy was inhibited. The results indicated that the *SHDA* gene is necessary for TSLP-mediated mitophagy activation. We further examined the epigenetic status of the *SHDA* promoter to determine the mechanism of TSLP-induced *SDHA* expression. The data showed that TSLP-induced *SHDA* expression was inhibited by the HAT inhibitor anacardic acid (AA) and the histone methyltransferase inhibitor methylthioadenosine (MTA) (Fig. 4A, B). These data indicated that histone acetylation and methylation were involved in TSLP-induced *SHDA* expression. Therefore, we examined acetylation at H3 and H4 sites and trimethylation at the H3K4, H3K9, and H3K36 sites of the *SHDA* promotor. The ChIP assay revealed that TSLP enhanced H3 acetylation and H4 acetylation in the *SHDA* promoter. Moreover, TSLP also significantly enhanced H3K4 and H3K36 trimethylation in the *SHDA* promoter (Fig. 4C–G).

### TSLP increased mitophagy-related protein expression via histone modification in THP-1 cells

**Fig. 5:**
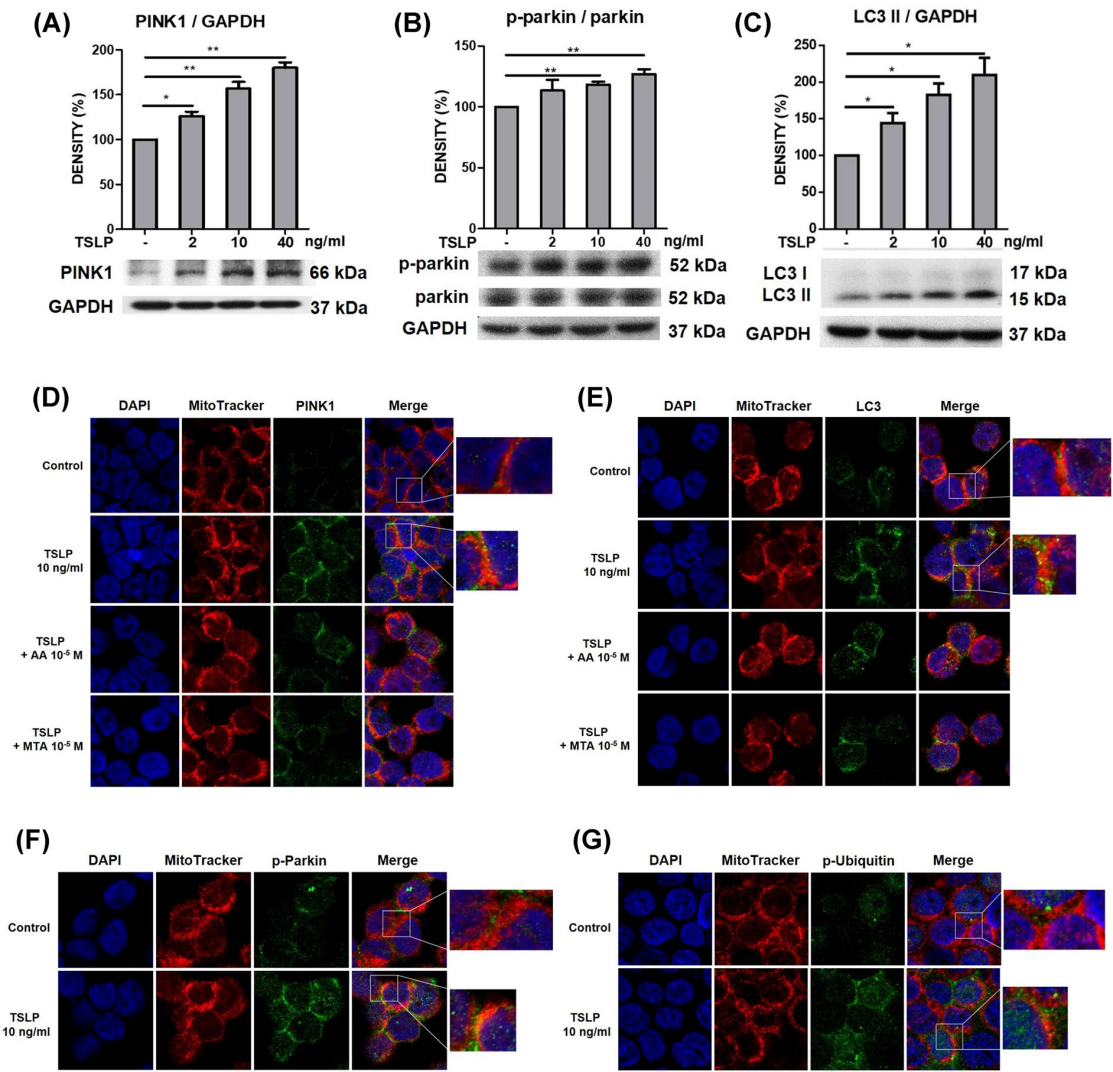
TSLP increased mitophagy-related protein expression in THP-1 cells via histone modification. The protein signal intensities of **A** PINK1, **B** phospho-Parkin (p-parkin), and **C** LC3 after treatment with different concentrations (0, 2, 10, 40 ng/mL) of TSLP for 8 h was determined by western blotting. The standard deviations of the optical density data were calculated from three independent experiments, and one representative result of the set of three is shown. *p < 0.05 and **p < 0.01 for the comparison with and without TSLP treatment. **D** Confocal laser microscopy showed that TSLP (10 ng/mL) treatment for 24 h significantly increased PINK1 signals and promoted PINK1 colocalization with mitochondria. Pretreatment with the histone acetyltransferase inhibitor AA and the histone methyltransferase inhibitor MTA reduced TSLP-induced PINK1 signals in mitochondria. **E** Confocal laser microscopy showed that TSLP (10 ng/mL) treatment for 24 h significantly increased LC3 signals and promoted LC3 colocalization with mitochondria. Pretreatment with AA and MTA reduced TSLP-induced LC3 signals in mitochondria, as determined by confocal laser microscopy. Confocal laser microscopy showed that TSLP (10 ng/mL) treatment for 24 h significantly increased **F** phosphor-parkin (p-Parkin) and **G** phospho-ubiquitin (p-Ubiquitin) signals and promoted mitophagy-related protein colocalization with mitochondria

Mitophagy often occurs in defective mitochondria following damage or stress. Owing to the finding of TSLP-induced ROS production and mitochondrial damage, we further examined whether TSLP could induce mitophagy-related protein (PINK1, Parkin, and LC3) signals in human monocytes. Western blot analysis showed that TSLP (2, 10 and 40 ng/mL) significantly increased the signal intensities of PINK1, phospho-Parkin and LC3 after TSLP treatment (Fig. 5A–C). Confocal laser microscopy showed that TSLP treatment increased PINK1 (Fig. 5D), LC3 (Fig. 5E), p-Parkin (Fig. 5F), and p-ubiquitin (Fig. 5G) accumulation in mitochondria, representing a typical phenomenon associated with mitophagy. Moreover, pretreatment with AA and MTA reduced TSLP-induced PINK1 and LC3 accumulation in mitochondria (Fig. 5D, E). These data indicated that histone acetylation and methylation regulate TSLP-induced mitophagy. We also examined another mitophagy-related pathway, hypoxia-inducible factor (HIF)-1-BCL2-interacting protein 3 (BINP3) signaling. The results showed that the signal intensities of HIF-1α, BINP3, and BNIP3 L/NIX was not activated by TSLP treatment (Additional file 2: Fig. S2).

### TSLP regulated mitochondrial biogenesis and promoted mitophagy via AMPK activation

**Fig. 6:**
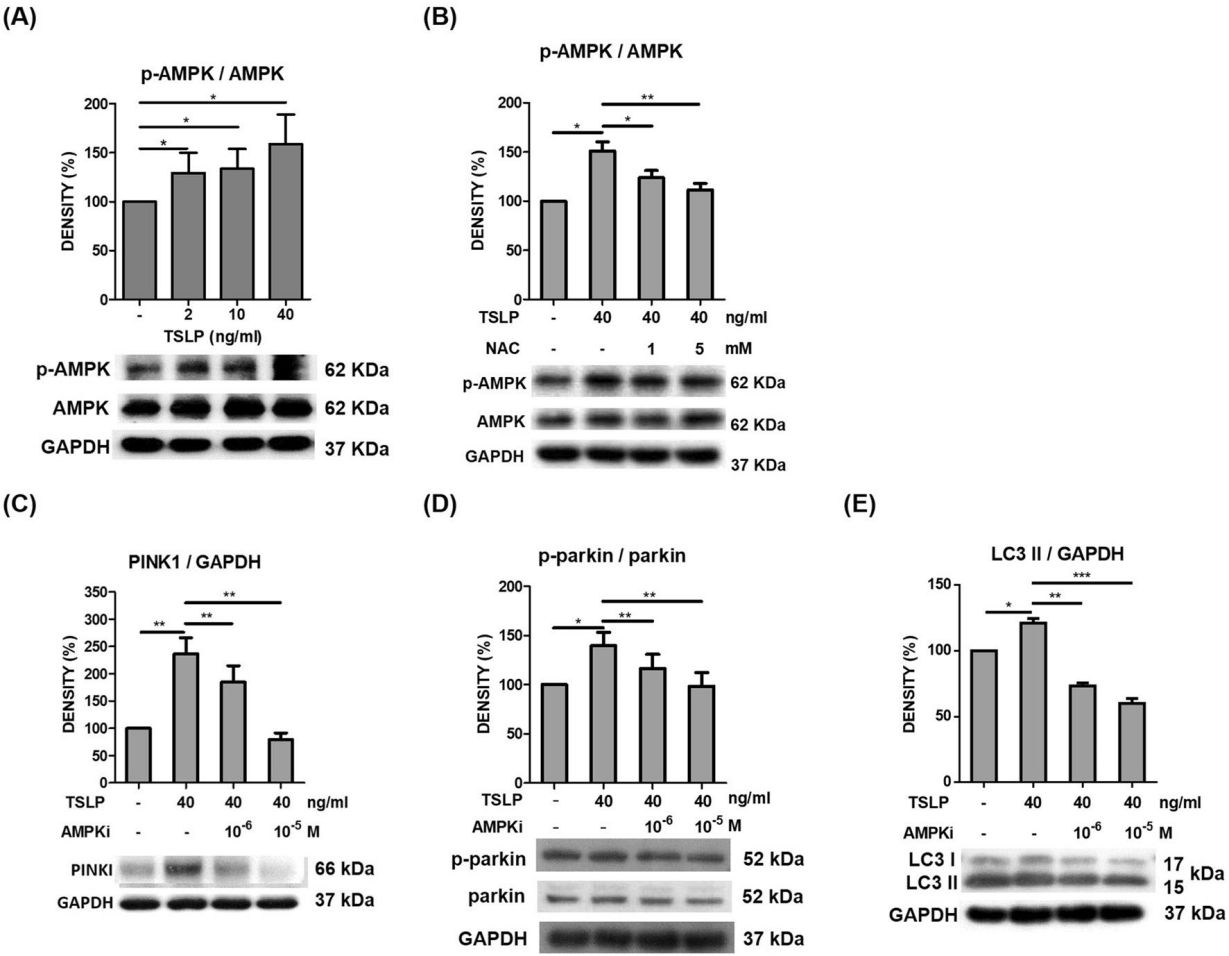
AMPK activation promoted TSLP-induced mitophagy-related protein expression. **A** TSLP (2, 10 and 40 ng/mL) significantly increased phospho-AMPK (p-AMPK) signal intensity, as shown by western blot analysis. **B** TSLP-induced p-AMPK signal intensity was suppressed by the antioxidant N-acetylcysteine (NAC). TSLP-induced protein signal intensities of **C** PINK1, **D** phospho-Parkin (p-Parkin), and **E** LC3 were suppressed by an AMPK inhibitor, as shown by western blot analysis. The standard deviations of the optical density data were calculated from three independent experiments, and one representative result of the set of three is shown. *p < 0.05, **p < 0.01 and ***p < 0.001

AMPK is a critical sensor of cellular energy status and has been reported to mediate mitophagy and promote mitochondrial fission [20]. Some studies have reported that AMPK activates both PINK1 and parkin phosphorylation [21, 22]. Thus, we examined whether TSLP-induced mitophagy is mediated by AMPK signaling and interplays with PINK1-Parkin signaling. Western blot analysis showed that TSLP significantly increased phospho-AMPK signal intensity (Fig. 6A), and the effect was inhibited by the antioxidant NAC (Fig. 6B). Moreover, TSLP-induced PINK, phospho-Parkin and LC3 signal intensity was decreased by dorsomorphin, an AMPK inhibitor (AMPKi), as shown by western blot analysis (Fig. 6C–E). These data indicated that TSLP regulated AMPK activation, which medicated mitochondrial biogenesis and promoted mitophagy-related protein signaling.

### TSLP-induced mitophagy shifted M1/M2 chemokine expression

**Fig. 7:**
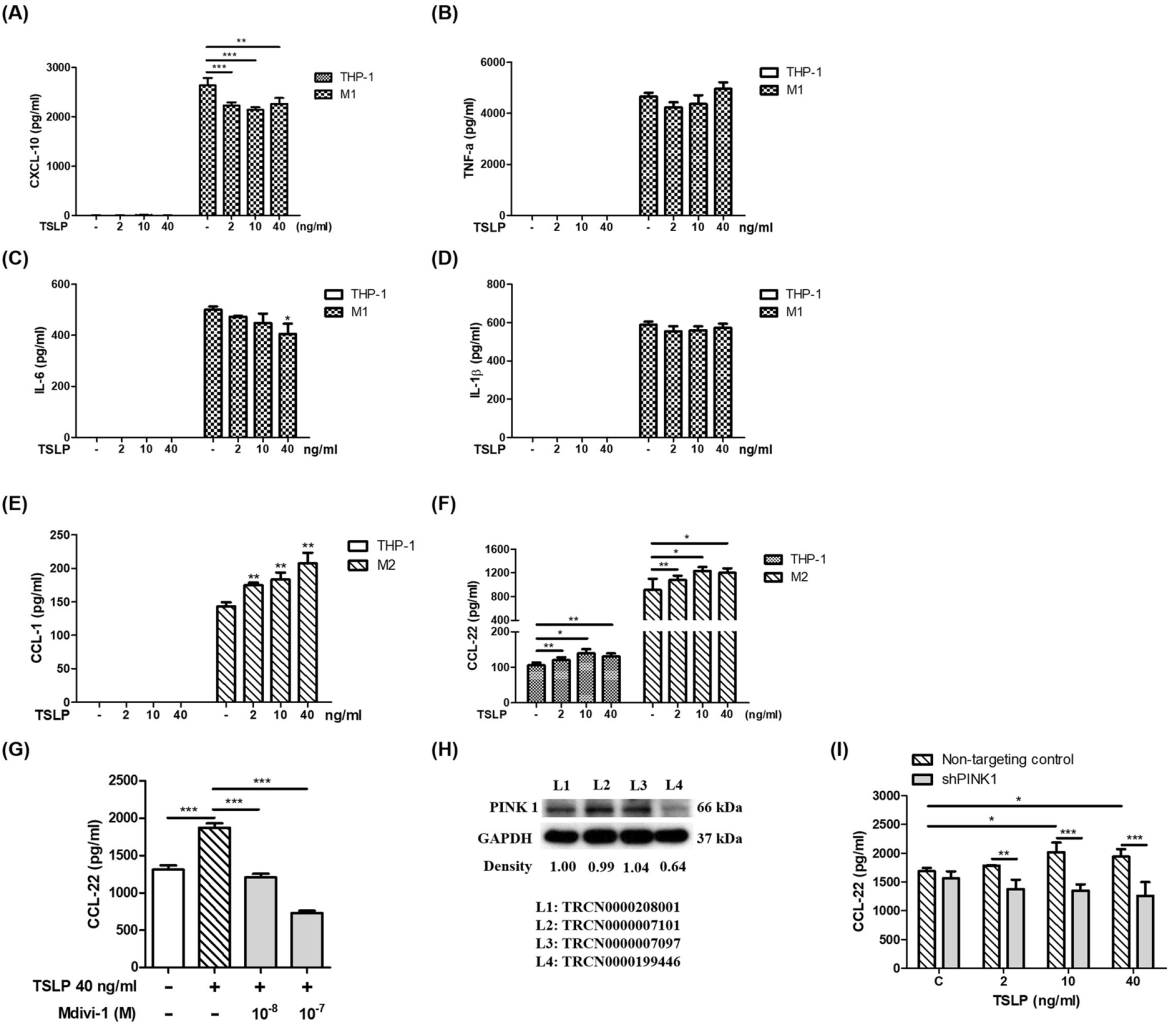
TSLP-induced mitophagy shifted M1/M2 chemokine expression. The M1-related chemokines **A** CXCL-10, **B** TNF-α, **C** IL-6, and **D** IL-1β in the cell culture medium of THP-1 cells and THP-1-derived M1 cells with or without TSLP treatment for 48 h were measured by ELISA. The M2-related cytokines **E** CCL-1 and **F** CCL22 in the cell culture medium of THP-1 cells and THP-1-derived M2 cells with or without TSLP treatment for 48 h were measured by ELISA. **G** The concentrations of CCL-22 in the culture medium of THP-1 cells and THP-1-derived M2 cells with or without pretreatment with a mitochondrial division inhibitor (mdivi-1) and 48 h of TSLP treatment were measured by ELISA. **H** The knockdown efficiency of PINK1 was determined by western blot. **I** The CCL-22 concentrations in the cell culture medium of THP-1 cells and THP-1-derived M2 cells with or without PINK1 knockdown and 48 h of TSLP treatment were measured by ELISA. Each bar represents the mean ± standard deviation of three independent experiments. *p < 0.05, **p < 0.01 and ***p < 0.001

To investigate the functional effect of TSLP-induced mitophagy on human monocytes, we examined the effects of TSLP on chemokine production. TSLP significantly decreased the production of the M1-related chemokine CXCL-10 (Fig. 7A) and cytokine IL-6 (Fig. 7C) but did not affect TNF-α (Fig. 7B) or IL-1β (Fig. 7D) production. TSLP significantly increased the production of the M2-related cytokine CCL-1 and chemokine CCL-22 (Fig. 7E, F). These data indicated that TSLP promoted M2 macrophage polarization. Moreover, TSLP-induced CCL-22 expression was suppressed by the mitophagy inhibitor Mdivi-1 and PINK1 gene knockdown (Fig. 7G, I). The PINK1 knockdown efficiency is shown in Fig. 7H. We used TRCN0000199446 to investigate the effects of PINK1 on the TSLP-induced CCL-22 concentration of M2 macrophages. These data indicated that TSLP-induced mitophagy mediated M1/M2 chemokine expression. TSLP-induced mitophagy was associated with a shift in macrophage M1/M2 polarization.

## Discussion

TSLP, IL-33 and IL-25 are epithelial cell-derived cytokines that stimulate group 2 innate lymphoid cells (ILC2s) to release type 2 cytokines such as IL-5 and IL-13 and play an important role in asthma pathogenesis [23]. It has been reported that rhinovirus (RV) infection of respiratory epithelial cells leads to TSLP production, which recruits ILC2s that amplify inflammation and promote airway fibrosis [24]. RV infection in allergic mice induces neutrophilic exacerbation and increases lung TSLP exclusively at sites of inflammation [25]. RV infection has been suggested to be associated with the development of asthma, and RV16 infection upregulates TSLP and IL-33 expression in human lung epithelial cells [26]. In the present study, TSLP induced ROS production, suggesting that RV may further induce ROS production and associated inflammation via the induction of TSLP.

Inflammatory cells that are recruited to the asthmatic airway have an exceptional capacity to produce a variety of ROS, contributing to tissue damage and airway inflammation [8]. Environmental factors, including ozone, diesel exhaust particles, tobacco smoke and respiratory virus infections, increase the levels of cellular ROS and induce mitochondrial dysfunction in the airway epithelium, which exacerbates allergic inflammation in asthma [27]. The responses of Th2-like cytokines, such as TSLP, to ROS and the electron transport chain have yet to be assessed. In the present study, we found that TSLP could induce marked ROS production, which is an important factor in the pathophysiology of asthma. Furthermore, we also provided an understanding of the mechanisms underlying TSLP-induced ROS production. In the present study, TSLP promoted complex I and II/III activity, suggesting that mitochondria were involved in TSLP-induced ROS production. Recently, complex I and III redox centers have been implicated as the major sites of mitochondrial ROS production, and complex II is also capable of producing ROS [28, 29]. UCPs are members of the anion carrier protein family on the inner mitochondrial membrane and cannot transport protons in the absence of specific activators; however, UCPs transport protons and increase the net proton conductance of mitochondria in the presence of specific activators, including ROS [30, 31]. We found that mitochondrial copy number, UCPs and complex II *SDHA* gene expression were significantly increased by TSLP. These data indicated that TSLP regulated mitochondrial redox in human monocytes.

Another novel finding of the present study is the epigenetic regulation of TSLP-induced ROS production. Histone acetylation and trimethylation, which are usually carried out by a variety of HATs or methyltransferases, are associated with gene activation [17]. In this study, TSLP increased H3 acetylation and H3K4 and H3K36 trimethylation in the complex II *SHDA* gene promoter. Confocal laser microscopy showed that pretreatment with AA and MTA also reduced TSLP-induced PINK1 and LC3 expression in mitochondria. These findings suggest that epigenetic regulation may be an important mechanism by which TSLP induces ROS production and mitophagy.

TSLP shifts macrophage polarization to an M2-like phenotype [32]. According to the concept of classic (M1) and alternatively (M2) activated macrophages, M1 macrophages are very sensitive to excessive ROS because of their high oxidative stress response [33]. In contrast, M2 macrophages inhibit ROS through metabolic regulation [34]. In the present study, we found that TSLP shifted M1/M2 chemokine expression. Our data suggest that TSLP promotes M2-like effects in a human monocyte cell line. Based on these M1/M2 characteristics in response to ROS, inflammatory M1 macrophages may be sensitive to TSLP-induced ROS-mediated injury (mitophagy/apoptosis), and allergic M2 macrophages may inhibit TSLP-induced ROS-mediated injury, thereby altering macrophage polarization toward allergic phenotypes.

Mitochondria not only play a vital metabolic role but also cause cell death via toxic products associated with oxidative phosphorylation and mitochondrial quality control at the protein, organelle and suborganelle levels [35]. Mitophagy is a selective form of autophagy that eliminates dysfunctional mitochondria to maintain overall mitochondrial health. Mitophagy plays an important role in supporting the immune system. Increased mitophagy is a component of the lung response to inflammation [36, 37]. AMPK, a master energy-sensing kinase, is activated in response to rising AMP levels following ATP hydrolysis and is a guardian of metabolism and mitochondrial homeostasis [20, 38]. It has been reported that PINK1 can be induced by TGF-β1 in pulmonary fibrosis [10]. In the present study, we found that TSLP clearly induced ROS production and subsequently increased the signals of the mitophagy-associated proteins PINK1, Parkin and LC3 through the AMPK signaling pathway. Our findings suggested that TSLP-induced ROS production and mitophagy in monocytes-macrophages may be associated with pulmonary fibrosis in asthma.

## Conclusion

**Fig. 8:**
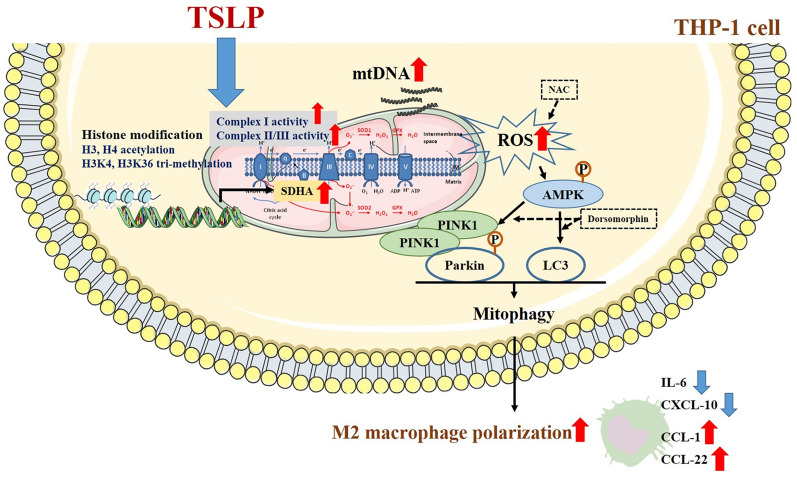
The proposed intracellular mechanisms underlying TSLP-mediated regulation of ROS production and mitophagy in human monocytes. TSLP increased ROS production, regulated mitochondrial biogenesis and mitophagy, and then shifted M1/M2 chemokine expression via the AMPK signaling pathway and histone modification in human monocytes

TSLP, a Th2-like cytokine, plays an important role in the pathogenesis of asthma, allergies, and other airway diseases. Understanding the possible mechanisms of the effect of TSLP on ROS production can help us identify targets for therapy or find novel pharmaceutical strategies to reduce asthma morbidity. All the findings in the present study suggest that epithelial-derived TSLP may induce not only Th2-related cytokine expression but also ROS production, subsequently influencing cellular oxidative stress and mitochondrial quantity, regulating mitophagy and shifting M1/M2 chemokine expression through the AMPK signaling pathway and histone modification in monocytes (Fig. 8). These findings broaden the knowledge of the effects of TSLP on ROS-related mitophagy and chemokine expression in allergic and inflammatory diseases.

## Methods

### Cell culture and measurement of ROS production

THP-1 cells (American Type Culture Collection, Rockville, MD, USA) were cultured in RPMI-1640 medium (Gibco, Carlsbad, CA, USA). The cells were resuspended in fresh media at a concentration of 1 × 10^5^/mL and pretreated with or without TSLP (PEPROTECH, New Jersey, USA) for 2 h. ROS production was measured with a dichlorodihydrofluorescein diacetate (DCFH-DA, Sigma–Aldrich, MO, USA) assay by flow cytometry according to the methods described in previous studies [39, 40]. Following treatment with TSLP, THP-1 cells were probed with 1 mM DCFH-DA and then washed with PBS once for immediate detection of dichlorofluorescein (DCF).

### Mitochondrial complex activity

The mitochondrial fraction was isolated from THP-1 cells using a mitochondrial isolation kit, and the activities of complex I and complex II/III in whole cell lysates or isolated mitochondria were measured using a microplate assay kit (Abcam, Cambridge, MA, USA) according to the manufacturer’s instructions.

### Measurement of ΔψM and transmission electron microscopy

THP-1 cells were treated with TSLP for 8 h, and JC-1 dye (Invitrogen, Waltham, USA) was used to measure the mitochondrial membrane potential by LSR II flow cytometry (Becton Dickinson, San Jose, CA). JC-1 dye exhibits potential-dependent accumulation in mitochondria, indicated by a fluorescence emission shift from green (525 nm) to red (590 nm). Mitochondrial depolarization is indicated by a decrease in the red/green fluorescence intensity ratio. After TSLP treatment, the cells were prepared for JEM-2000EXII transmission electron microscopy (JEOL Ltd. Tokyo, Japan) to observe the morphology of mitochondria and mitophagy.

### Preparation of mitochondrial DNA (mtDNA) and relative quantification of mtDNA fragments

The total mtDNA of THP-1 cells were isolated after 8-h TSLP stimulation by a mitochondrial DNA isolation kit (BioVision, Milpitas, USA) following the manufacturer’s instructions. Two primer sets specific for the mitochondrial ribosomal 16SRNA, mtDNA-79 and mtDNA-230, were designed as described in a previous study [41, 42]. The 79-bp fragment (mtDNA-79) represents mitophagy-mediated mtDNA generated by enzymatic cleavage in apoptotic cells. In contrast, the 230-bp fragment (mtDNA-230) corresponds to mtDNA released by non-apoptotic types of cell death (i.e., necrosis) or active secretion [41–43]. Quantitative analysis of mtDNA fragments was performed by quantitative real-time PCR, and the data were evaluated for mtDNA fragments relative to housekeeping gene (ACTB) expression.

### Mitophagy assay

To detect mitophagy, we used the Mitophagy Detection Kit (Dojindo Molecular Technologies, Inc., Rockville, MD, USA) according to the manufacturer’s instructions. Briefly, THP-1 cells were incubated with serum-free RPMI containing 100 nM mitophagy dye at 37 °C for 30 min. After washing and discarding the supernatant, the cells were stimulated with 40 ng/mL TSLP in complete medium for 12 h. Mitophagy dye fluorescence was detected by an LSR II flow cytometer (Becton Dickinson, San Jose, CA) with a PerCP Cy5.5 channel. The data were analyzed using FCS Express 4 Flow Research (De Novo Software, California, USA).

### Quantitative real-time reverse transcription polymerase chain reaction (qRT–PCR) analysis of respiratory chain and mitochondrial biogenesis factors

The gene expression of mitochondrial copy numbers, respiratory chain complexes I–V, including NADH dehydrogenase, subunit 1 (*MT-ND1,* MT complex I), succinate dehydrogenase complex, subunit A, flavoprotein (*SDHA,* MT complex II), mitochondrial cytochrome b (*MT-CYTB,* MT complex III), cytochrome c oxidase I (*COXI,* MT complex IV), ATP synthase, H + transporting, mitochondrial Fo complex, subunit F6 (*MT-ATP6,* MT complex V), and mitochondrial proton carrier: uncoupling protein 2 (*UCP2*) were detected to investigate mitochondrial involvement. After treatment with TSLP for different durations, total RNA (1 μg) was reverse transcribed by SuperScript II using an anchored oligo-dT primer as instructed by the manufacturer (Invitrogen, Carlsbad, CA, USA), and the resulting DNA was used as template for qRT–PCR. The primer sequences and qRT–PCR were performed as described in our previous work [44, 45].

### Chromatin immunoprecipitation (ChIP) assay

ChIP was performed as described previously with minor modifications [46]. Primers and probes were designed to analyze the proximal promoter regions relative to the transcription start site of the *SDHA* gene (− 2975/− 2577, including the AP-1 binding site) according to MatInspector (Genomatix Software, München, Germany). The primer sequences were as follows: forward: 5′-CTGACCACACTACCTCAGCA and reverse: 5′-CAGTGCTTGCTTCTTGGTGA. All TaqMan reagents were purchased from Applied Biosystems. The relative intensities of the amplified products were normalized to the input DNA levels.

### Western blotting

THP-1 cells were treated with TSLP for 2, 4, 6 or 8 h and analyzed by western blotting with anti-MTCO2 antibody (Abcam), anti-5′-AMP-activated protein kinase (anti-AMPK, Cell Signaling, Massachusetts, USA), anti-phospho-AMPK (Cell Signaling Technology, Danvers, MA, USA), anti-PINK1 (Cell Signaling Technology, Danvers, MA, USA), anti-LC3 (MBL, Nagoya, Japan), anti- phospho-Parkin (Ser65) Antibody #36,866 (Cell Signaling Technology, Danvers, MA, USA) and anti-GAPDH antibodies (Santa Cruz Biotechnology, Santa Cruz, CA, USA). Western blotting analysis was performed as described in our previous work [46].

### Immunofluorescence staining

Cultured THP-1 cells were treated with or without TSLP and fixed on glass slides. The cell slides were washed twice with PBS and permeabilized with 0.5% Triton for 5 min. These slides were then incubated in blocking solution (5% normal goat serum and 1% bovine serum albumin in PBS) for 30 min. After being blocked, the slides were incubated with PINK1, LC3, phospho-Parkin (Ser65) Antibody #36,866 (Cell Signaling Technology, Danvers, MA, USA) or phospho-Ubiquitin (Ser65), Cat. No. ABS1513-I (Merck Millipore, Darmstadt, Germany) antibodies, followed by Alexa Fluor 488-labeled and Alexa Fluor 568-labeled MitoTracker mitochondrial tracking dye for 1 h. After washing, 4′,6′-diamidino-2-phenylindole (DAPI) (300 nM; Invitrogen) dissolved in PBS was used as a nuclear staining dye, and the slides were mounted on Vectashield (Vector Laboratories Inc.), covered with glass coverslips, and imaged using an LSM 700 microscope (Carl Zeiss Microscopy, Gottingen, Germany).

### Enzyme-linked immunosorbent assay (ELISA)

After 2 h of TSLP pretreatment, THP-1 cells were exposed to phorbol 12-myristate 13-acetate (PMA, 10 ng/mL, Sigma–Aldrich), LPS (10 ng/mL, Sigma–Aldrich) and IFN-γ (10 ng/mL, PEPROTECH) to induce M1 polarization or exposed to PMA and IL-4 (10 ng/mL, PEPROTECH) to induce M2 polarization. After being treated with or without PMA/LPS/IFN-γ or PMA/IL-4, the cell-free media were collected at 48 h, and CXCL10, TNF-α, IL-6, IL-1β, CCL-1, and CCL-22 were measured by ELISA (R & D Systems, Minneapolis, MN). CCL-22 concentrations in THP-1 cells and PMA/IL-4-primed THP-1 cells with or without the mitophagy inhibitor Mdivi-1 were also analyzed by ELISA.

### Gene knockdown

THP-1 cells were transduced using shRNA lentiviral particles at a multiplicity of infection (MOI) of 1. A MOI is the number of transducing lentiviral particles per cell. All shRNA lentiviral particles were purchased from the RNA technology platform and gene manipulation core of Academia Sinica. The lentivirus encoded either a nontargeted (nt) shRNA (TRCN0000208001) or a shRNA directed against PINK1 (#TRCN0000007101, #TRCN0000007097, and #TRCN0000199446). A nontargeting (nt) shRNA (#TRCN0000208001) or shRNA targeting *SHDA* (#TRCN0000028085, #TRCN0000028093, and #TRCN000028118) was used to investigate whether *SHDA* was involved in TSLP-induced mitophagy. PINK1-knockdown cells were pretreated with TSLP for 2 h and then polarized to form M2 macrophages with PMA (20 ng/mL) and IL-4 (20 ng/mL). The supernatants were collected, and the CCL-22 concentration was measured after 48 h by ELISA.

### Statistical analysis

GraphPad Prism (Version 5, GraphPad Prism Software, Los Angeles, CA, USA) and IBM SPSS statistical software (Version 19, IBM Company, Armonk, NY, USA) were used for statistical analysis. The Mann–Whitney U test was used for pairwise comparisons between samples with and without TSLP treatment. The immunoreactivities of mitophagy-related proteins in western blot analysis were normalized to those of the total proteins. The western blot results were analyzed by the Wilcoxon signed-rank test. Multiple nonparametric comparisons to assess the effects of TSLP on chemokines were analyzed by the Kruskal–Wallis test with post hoc analysis. Statistically significant differences were considered if a *p* value < 0.05.

## Supplementary Information

**Additional file 1: Figure S1.** Respiratory chain complex II SHDA *is* involved in TSLP-induced mitophagy. **A** The knockdown efficiency of SHDA was determined by western blot. **B** Mitophagy detection dye to quantify THP-1 cell mitophagy showed significantly increased fluorescence upon treatment with 40 ng/mL TSLP in the nontargeting control. When *SHDA* was knocked down, the increased fluorescence intensity of TSLP-induced mitophagy was inhibited. *p < 0.05, **p < 0.01 and ***p < 0.001 between THP-1 cells with and without TSLP treatment.

**Additional file 2: Figure S2.** The effects of TSLP on hypoxia-inducible factor (HIF)-1-BCL2-interacting protein 3 (BINP3) signaling.

## Data Availability

The data presented in this study are available on request from the corresponding author. Data may be available upon request to interested researchers. Please send data requests to Chih-Hsing Hung, MD, PhD. Department of Pediatrics, Kaohsiung Medical University Hospital, Kaohsiung Medical University.

## Abbreviations

AA: Anacardic acid
AMPK: 5′-AMP-activated protein kinase
AMPKi: AMPK inhibitor
ATP6: ATP synthase, H + transport, mitochondrial Fo complex, subunit F6
BINP3: BCL2-interacting protein 3
COXI: Cytochrome c oxidase I
ChIP: Chromatin immunoprecipitation
CYTB: Mitochondrial cytochrome b
DAPI: 4′,6′-Diamidino-2-phenylindole
DCF: Dichlorofluorescein
DCFH-DA: Dichloro-dihydro-fluorescein diacetate
ELISA: Enzyme-linked immunosorbent assay
HIF: Hypoxia-inducible factor
HAT: Histone acetyltransferase
HDAC: Histone deacetylase
H3: Histone 3
IL: Interleukin
ILC2: Group 2 innate lymphoid cells
LC3: Light-chain 3
MOI: Multiplicity of infection
MTA: Methylthioadenosine
MTCO2: Mitochondrial inner membrane protein
mtDNA: Mitochondria DNA
NAC: N-acetylcysteine
ND1: NADH dehydrogenase, subunit 1
PINK1: PTEN-induced putative kinase 1
PMA: Phorbol 12-myristate 13-acetate
qRT–PCR: Quantitative real-time reverse transcription polymerase chain reaction
ROS: Reactive oxygen species
RV: Rhinovirus
SDHA: Succinate dehydrogenase complex, subunit A, flavoprotein
TEM: Transmission electron microscopy
TSLP: Thymic stromal lymphopoietin
UCPs: Uncoupling proteins

## References

[1] Saenz SA, Taylor BC, Artis D (2008). Welcome to the neighborhood: epithelial cell-derived cytokines license innate and adaptive immune responses at mucosal sites. Immunol Rev.

[2] Ziegler SF (2012). Thymic stromal lymphopoietin and allergic disease. J Allergy Clin Immunol.

[3] Ito T, Wang YH, Duramad O, Hori T, Delespesse GJ, Watanabe N (2005). TSLP-activated dendritic cells induce an inflammatory T helper type 2 cell response through OX40 ligand. J Exp Med.

[4] Lin SC, Huang JJ, Wang JY, Chuang HC, Chiang BL, Ye YL (2016). Upregulated thymic stromal lymphopoietin receptor expression in children with asthma. Eur J Clin Invest.

[5] Gluck J, Rymarczyk B, Kasprzak M, Rogala B (2016). Increased levels of interleukin-33 and thymic stromal lymphopoietin in exhaled breath condensate in chronic bronchial asthma. Int Arch Allergy Immunol.

[6] Ferreira DS, Annoni R, Silva LF, Buttignol M, Santos AB, Medeiros MC (2012). Toll-like receptors 2, 3 and 4 and thymic stromal lymphopoietin expression in fatal asthma. Clin Exp Allergy.

[7] Harada M, Hirota T, Jodo AI, Hitomi Y, Sakashita M, Tsunoda T (2011). Thymic stromal lymphopoietin gene promoter polymorphisms are associated with susceptibility to bronchial asthma. Am J Respir Cell Mol Biol.

[8] Zuo L, Otenbaker NP, Rose BA, Salisbury KS (2013). Molecular mechanisms of reactive oxygen species-related pulmonary inflammation and asthma. Mol Immunol.

[9] Zuo L, Nogueira L, Hogan MC (2011). Reactive oxygen species formation during tetanic contractions in single isolated *Xenopus* myofibers. J Appl Physiol.

[10] Patel AS, Song JW, Chu SG, Mizumura K, Osorio JC, Shi Y (2015). Epithelial cell mitochondrial dysfunction and PINK1 are induced by transforming growth factor-beta1 in pulmonary fibrosis. PloS ONE.

[11] Lee IT, Yang CM (2012). Role of NADPH oxidase/ROS in pro-inflammatory mediators-induced airway and pulmonary diseases. Biochem Pharmacol.

[12] Youle RJ, Narendra DP (2011). Mechanisms of mitophagy. Nat Rev Mol Cell Biol.

[13] Pickles S, Vigié P, Youle RJ (2018). Mitophagy and quality control mechanisms in mitochondrial maintenance. Curr Biol.

[14] Pickrell AM, Youle RJ (2015). The roles of PINK1, parkin, and mitochondrial fidelity in Parkinson’s disease. Neuron.

[15] Tanida I, Ueno T, Kominami E (2004). LC3 conjugation system in mammalian autophagy. Int J Biochem Cell Biol.

[16] Larson-Casey JL, Deshane JS, Ryan AJ, Thannickal VJ, Carter AB (2016). Macrophage Akt1 kinase-mediated mitophagy modulates apoptosis resistance and pulmonary fibrosis. Immunity.

[17] Berger SL (2007). The complex language of chromatin regulation during transcription. Nature.

[18] DeVries A, Vercelli D (2015). Epigenetics in allergic diseases. Curr Opin Pediatr.

[19] Sabounchi S, Bollyky J, Nadeau K (2015). Review of environmental impact on the epigenetic regulation of atopic diseases. Curr Allergy Asthma Rep.

[20] Seabright AP, Fine NHF, Barlow JP, Lord SO, Musa I, Gray A (2020). AMPK activation induces mitophagy and promotes mitochondrial fission while activating TBK1 in a PINK1-Parkin independent manner. FASEB J.

[21] Wang B, Nie J, Wu L, Hu Y, Wen Z, Dong L (2018). AMPKα2 protects against the development of heart failure by enhancing mitophagy via PINK1 phosphorylation. Circ Res.

[22] Lee SB, Kim JJ, Han SA, Fan Y, Guo LS, Aziz K (2019). The AMPK-Parkin axis negatively regulates necroptosis and tumorigenesis by inhibiting the necrosome. Nat Cell Biol.

[23] Froidure A, Shen C, Gras D, Van Snick J, Chanez P, Pilette C (2014). Myeloid dendritic cells are primed in allergic asthma for thymic stromal lymphopoietin-mediated induction of Th2 and Th9 responses. Allergy.

[24] Kumar RK, Foster PS, Rosenberg HF (2014). Respiratory viral infection, epithelial cytokines, and innate lymphoid cells in asthma exacerbations. J Leukoc Biol.

[25] Mahmutovic-Persson I, Akbarshahi H, Bartlett NW, Glanville N, Johnston SL, Brandelius A (2014). Inhaled dsRNA and rhinovirus evoke neutrophilic exacerbation and lung expression of thymic stromal lymphopoietin in allergic mice with established experimental asthma. Allergy.

[26] Mehta AK, Duan W, Doerner AM, Traves SL, Broide DH, Proud D (2016). Rhinovirus infection interferes with induction of tolerance to aeroantigens through OX40 ligand, thymic stromal lymphopoietin, and IL-33. J Allergy Clin Immunol.

[27] Aguilera-Aguirre L, Bacsi A, Saavedra-Molina A, Kurosky A, Sur S, Boldogh I (2009). Mitochondrial dysfunction increases allergic airway inflammation. J Immunol.

[28] St-Pierre J, Buckingham JA, Roebuck SJ, Brand MD (2002). Topology of superoxide production from different sites in the mitochondrial electron transport chain. J Biol Chem.

[29] Quinlan CL, Orr AL, Perevoshchikova IV, Treberg JR, Ackrell BA, Brand MD (2012). Mitochondrial complex II can generate reactive oxygen species at high rates in both the forward and reverse reactions. J Biol Chem.

[30] Donadelli M, Dando I, Fiorini C, Palmieri M (2014). UCP2, a mitochondrial protein regulated at multiple levels. Cell Mol Life Sci.

[31] Considine MJ, Goodman M, Echtay KS, Laloi M, Whelan J, Brand MD (2003). Superoxide stimulates a proton leak in potato mitochondria that is related to the activity of uncoupling protein. J Biol Chem.

[32] Han H, Headley MB, Xu W, Comeau MR, Zhou B, Ziegler SF (2013). Thymic stromal lymphopoietin amplifies the differentiation of alternatively activated macrophages. J Immunol.

[33] O'Neill LA, Hardie DG (2013). Metabolism of inflammation limited by AMPK and pseudo-starvation. Nature.

[34] Pelegrin P, Surprenant A (2009). Dynamics of macrophage polarization reveal new mechanism to inhibit IL-1beta release through pyrophosphates. EMBO J.

[35] Parsons MJ, Green DR (2010). Mitochondria in cell death. Essays Biochem.

[36] Lazarou M (2015). Keeping the immune system in check: a role for mitophagy. Immunol Cell Biol.

[37] Chang AL, Ulrich A, Suliman HB, Piantadosi CA (2015). Redox regulation of mitophagy in the lung during murine *Staphylococcus**aureus* sepsis. Free Radical Biol Med.

[38] Herzig S, Shaw RJ (2018). AMPK: guardian of metabolism and mitochondrial homeostasis. Nat Rev Mol Cell Biol.

[39] Sanchez-Sanchez AM, Martin V, Garcia-Santos G, Rodriguez-Blanco J, Casado-Zapico S, Suarez-Garnacho S (2011). Intracellular redox state as determinant for melatonin antiproliferative vs cytotoxic effects in cancer cells. Free Radical Res.

[40] Zhao XY, Li GY, Liu Y, Chai LM, Chen JX, Zhang Y (2008). Resveratrol protects against arsenic trioxide-induced cardiotoxicity in vitro and in vivo. Br J Pharmacol.

[41] Budnik LT, Kloth S, Baur X, Preisser AM, Schwarzenbach H (2013). Circulating mitochondrial DNA as biomarker linking environmental chemical exposure to early preclinical lesions elevation of mtDNA in human serum after exposure to carcinogenic halo-alkane-based pesticides. PloS ONE.

[42] Jing R, Hu ZK, Lin F, He S, Zhang SS, Ge WY (2020). Mitophagy-mediated mtDNA release aggravates stretching-induced inflammation and lung epithelial cell injury via the TLR9/MyD88/NF-κB pathway. Front Cell Dev Biol.

[43] Lin JY, Jing R, Lin F, Ge WY, Dai HJ, Pan L (2018). High tidal volume induces mitochondria damage and releases mitochondrial DNA to aggravate the ventilator-induced lung injury. Front Immunol.

[44] Chen CH, Wang CZ, Wang YH, Liao WT, Chen YJ, Kuo CH (2014). Effects of low-level laser therapy on M1-related cytokine expression in monocytes via histone modification. Mediat inflamm.

[45] Lee CH, Wu SB, Hong CH, Liao WT, Wu CY, Chen GS (2011). Aberrant cell proliferation by enhanced mitochondrial biogenesis via mtTFA in arsenical skin cancers. Am J Pathol.

[46] Lin YC, Lin YC, Huang MY, Kuo PL, Wu CC, Lee MS (2017). Tumor necrosis factor-alpha inhibitors suppress CCL2 chemokine in monocytes via epigenetic modification. Mol Immunol.

