# Supplementary figures and images for: TSLP regulates mitochondrial ROS-induced mitophagy via histone modification in human monocytes
Source: Cell Biosci. 2022 Mar 15;12:32. doi: 10.1186/s13578-022-00767-w (PMC8925056; doi:10.1186/s13578-022-00767-w)

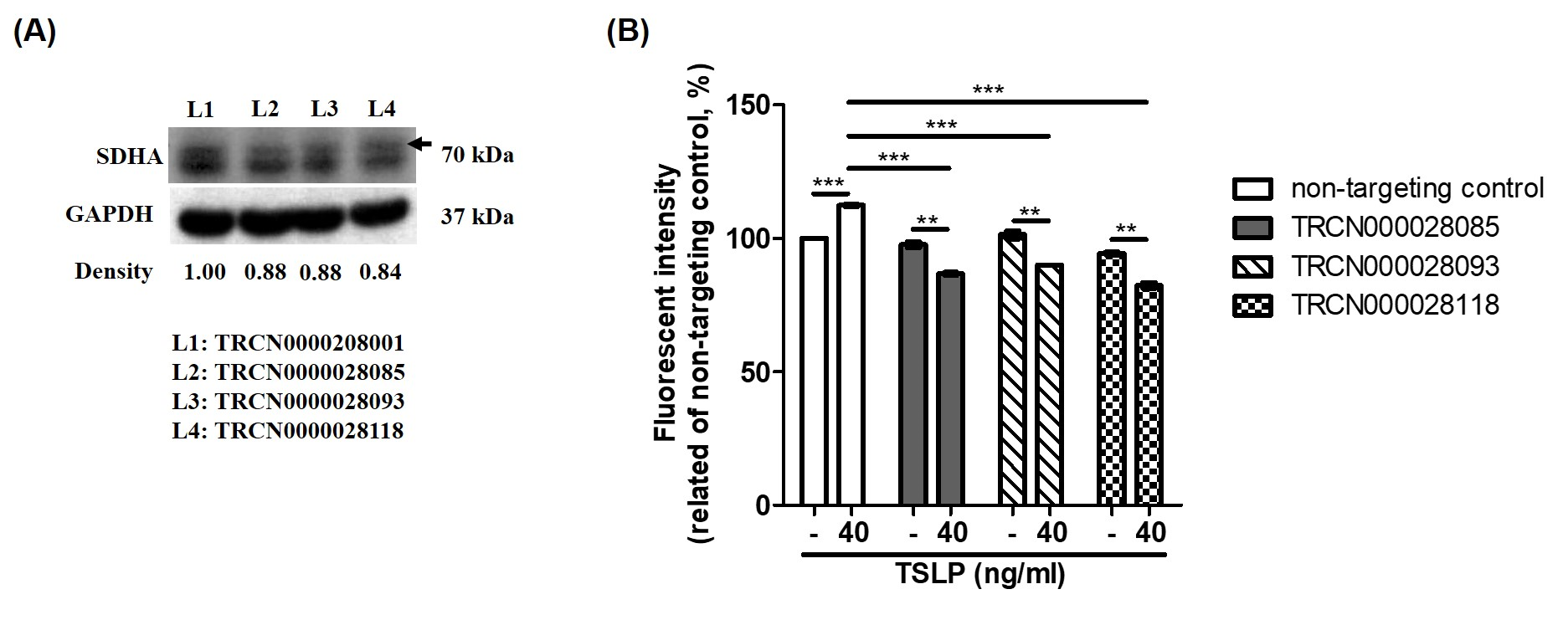

Supplement: Supplementary file 1 — Additional file 1: Figure S1. Respiratory chain complex II SHDA is involved in TSLP-induced mitophagy. A The knockdown efficiency of SHDA was determined by western blot. B Mitophagy detection dye to quantify THP-1 cell mitophagy showed significantly increased fluorescence upon treatment with 40 ng/mL TSLP in the nontargeting control. When SHDA was knocked down, the increased fluorescence intensity of TSLP-induced mitophagy was inhibited. *p < 0.05, **p < 0.01 and ***p < 0.001 between THP-1 cells with and without TSLP treatment. [file 13578_2022_767_MOESM1_ESM.tif]

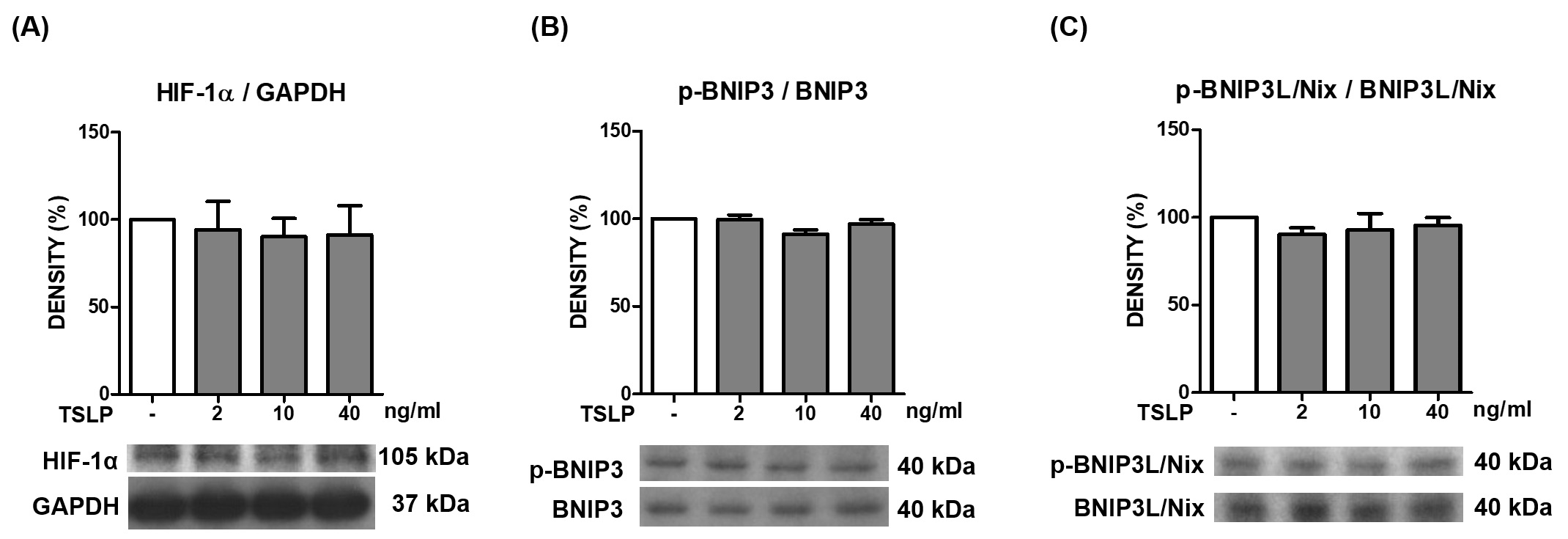

Supplement: Supplementary file 2 — Additional file 2: Figure S2. The effects of TSLP on hypoxia-inducible factor (HIF)-1-BCL2-interacting protein 3 (BINP3) signaling. [file 13578_2022_767_MOESM2_ESM.tif]
